# Research progress on the etiology and pathogenesis of pancreatic cancer: a narrative review

**DOI:** 10.3389/fonc.2026.1781024

**Published:** 2026-06-11

**Authors:** Meitong Guo, Qi Han, Zixi Wang, Dong Ya, Xiaoyan Wang

**Affiliations:** 1 The First Clinical Hospital of Jilin Academy of Traditional Chinese Medical Sciences, Changchun, China; 2 Capital Medical University, Beijing, China

**Keywords:** environmental factors, etiological mechanisms, genetic factors, pancreatic cancer, signaling pathways

## Abstract

Pancreatic cancer (PC) is a highly malignant tumor of the digestive system, characterized by the lack of effective methods for early screening, diagnosis and treatment. Despite its low incidence rate, it has an extremely high mortality rate. In recent years, the number of confirmed cases worldwide has been on a continuous rise, making PC the sixth leading cause of cancer-related deaths across the globe. At present, clinical treatment strategies including surgical resection, chemotherapy, radiotherapy, molecular targeted therapy and biological immunotherapy have not significantly improved the survival prognosis of PC patients. Therefore, exploring safe and effective therapeutic approaches and clarifying the etiological mechanisms of PC have become key priorities in clinical treatment. Its occurrence and progression involve multilayered mechanisms, including environmental exposure, genetic susceptibility, metabolic abnormalities, chronic inflammation, progression of precancerous lesions, remodeling of the tumor microenvironment, and immune escape. Based on a summary of smoking, alcohol consumption, obesity, diabetes, chronic pancreatitis, genetic mutations, and multiple signaling pathways, this article further provides a critical review from the perspectives of evidence strength, mechanistic plausibility, and clinical translational value. It aims to deepen the understanding of the initiation and progression of pancreatic cancer and to provide new directions for its early diagnosis and precision treatment.

## 1 Introduction

Pancreatic cancer (PC) is one of the most malignant gastrointestinal tumors, characterized by strong invasiveness and metastatic potential. It also features difficulties in early diagnosis and high malignancy, with a 5-year survival rate of merely 13% (1). The clinical manifestations of PC are relatively insidious, and there is currently a lack of clear early diagnostic strategies. Most patients are diagnosed at an advanced stage, over 80% of whom are deemed unresectable. Meanwhile, PC exhibits extensive resistance to radiotherapy and chemotherapy, and is not sensitive to immunotherapy, leading to suboptimal treatment outcomes and extremely poor prognosis (2). Over the past two decades, the annual number of PC-related deaths worldwide has surged from more than 200,000 to over 400,000, posing a severe threat to human health (3). Currently, PC has become one of the leading causes of cancer-related death worldwide. Regions with the highest incidence of PC include North America, Europe, and Australia, and the number of PC patients is projected to increase by more than 30% by 2040 (4). Approximately 60% of PC patients are diagnosed at an advanced stage or with distant metastasis (5). The incidence and mortality of PC vary widely globally, with an overall 5-year survival rate of 2%–9% (6). Even after undergoing radical surgery, 70%–80% of PC patients still develop local recurrence or distant metastasis (7).

Owing to the pancreas’ special anatomical location, the early symptoms of PC are concealed and non-specific. Patients often present with digestive disorders, nausea, diarrhea and other symptoms, which are easily confused with those of common gastrointestinal diseases. As the disease progresses, PC may manifest characteristic symptoms; for instance, patients with pancreatic head cancer are prone to painless jaundice (8). Cancer of the pancreatic body and tail usually presents as persistent upper abdominal pain that radiates to the lumbodorsal region, accompanied by significant weight loss and steatorrhea (9). When most PC patients show typical symptoms, the disease has mostly progressed to the middle or advanced stage. As a fatal malignant tumor, PC occurs predominantly in males, with the age of onset mainly concentrated at 40–85 years. It ranks first among asymptomatic cancers; 90% of patients have adenocarcinoma, and 10% exhibit a familial predisposition (10). Given the occult nature of early-stage PC and the limited efficacy of systemic therapy, merely emphasizing the disease burden of PC is no longer sufficient to support the novelty of an etiological review. A more valuable focus should shift toward the progression of precancerous lesions, feasible detection approaches, and the mechanisms underlying immunotherapy. Therefore, identifying safe and effective therapeutic strategies for PC remains an urgent clinical challenge.

**Figure 1:**
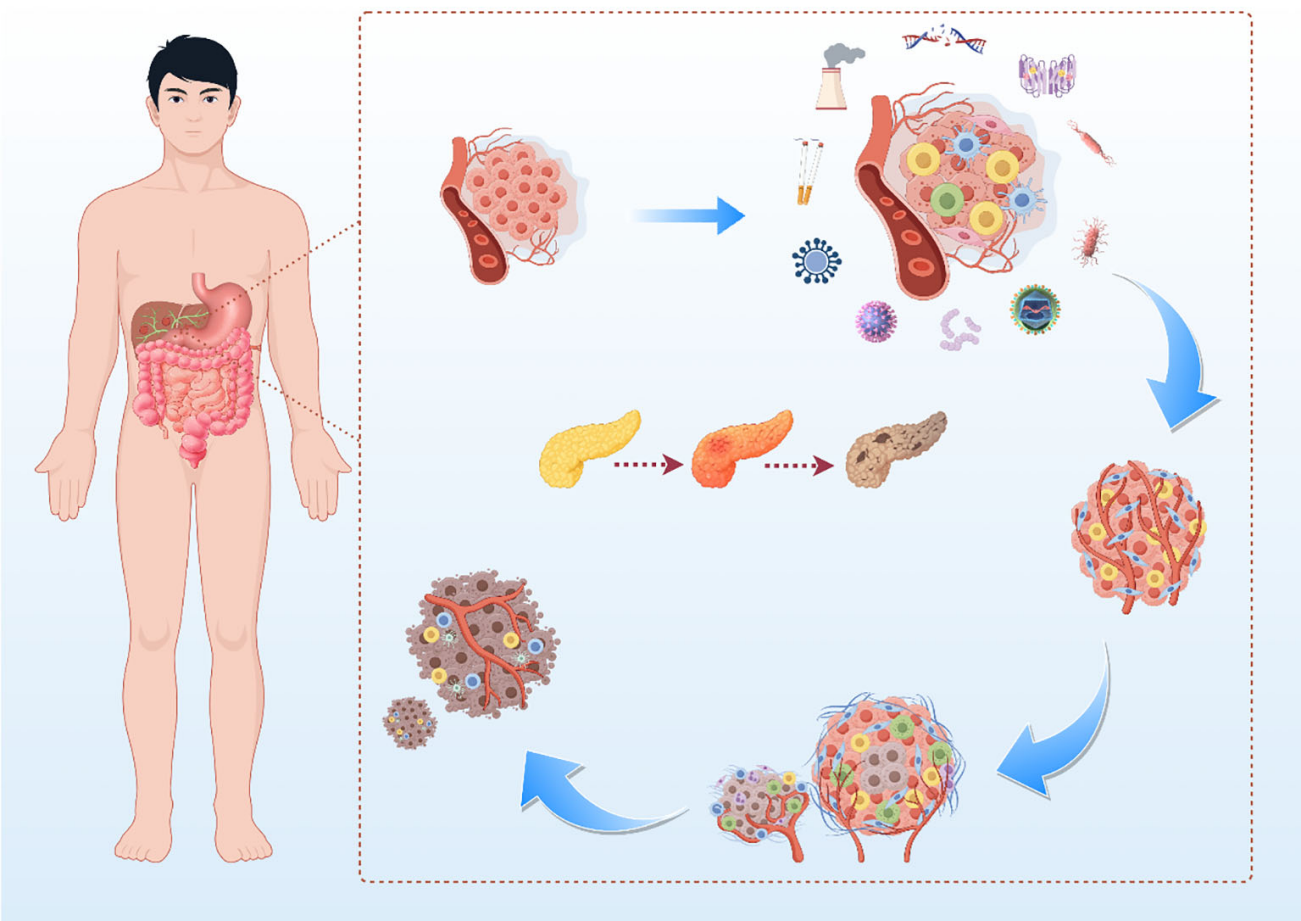
The pathogenesis of pancreatic cancer is relatively complex, which is closely associated with environmental factors, genetic factors, and other elements. During the pathogenesis of pancreatic cancer, a variety of pathological factors induce the transformation of normal pancreatic cells into a pattern of pancreatic epithelial or ductal cell dysplasia, pancreatic carcinoma *in situ*, and malignant invasive carcinoma.

At present, the etiology and pathogenesis of PC remain unclear. Existing studies mostly suggest that it is closely associated with environmental factors (11), genetic factors (12), as well as diseases such as diabetes mellitus (13) and chronic pancreatitis (14). Early molecular events represented by KRAS mutations can induce acinar-to-ductal metaplasia (ADM) and the formation of PanIN, while alterations in CDKN2A, TP53, and SMAD4 further drive the progression from high-grade intraepithelial neoplasia to invasive carcinoma (15). Therefore, simply listing epidemiological risk factors for PC is insufficient to explain its pathogenesis; a comprehensive analysis integrating pathological precursor lesions, driver genes, the inflammatory microenvironment, and histological sampling techniques is required. While summarizing the risk factors and core pathways of PC, this article focuses on distinguishing well-established drivers of PC, evidence with clinical translational relevance, and hypotheses that remain confined to experimental models, thereby enhancing the critical perspective and readability of the review (**Figure 1**).

## 2 Pathogenesis of pancreatic cancer

### 2.1 Precancerous lesions and models of early progression

In etiological studies of PC, the progression of precancerous lesions plays an important role. The major precursor lesions of pancreatic ductal adenocarcinoma (PDAC) include pancreatic intraepithelial neoplasia (PanIN), intraductal papillary mucinous neoplasm (IPMN), and mucinous cystic neoplasm (MCN) (16). Among these, PanIN is usually a microscopic lesion and often progresses along a trajectory from low-grade to high-grade lesions and subsequently to invasive carcinoma. In contrast, IPMN and MCN are cystic precursor lesions detectable by imaging, providing a more clearly defined window for clinical surveillance and intervention. Genomic studies have further demonstrated that IPMN and MCN can serve as direct precursors of invasive pancreatic cancer, and that there may be a clinically exploitable time window for early recognition and intervention between high-grade dysplasia and invasive carcinoma (17).

The PanIN progression model suggests that KRAS mutation is one of the earliest and most stable molecular events, as KRAS abnormalities can already be detected in low-grade lesions. In contrast, CDKN2A inactivation and alterations in TP53 and SMAD4 are more commonly observed at later stages, indicating that they are more likely involved in tumor progression and invasion rather than initiation alone (18). Therefore, although KRAS mutation itself can initiate aberrant differentiation programs, it is insufficient to explain the invasive behavior of all PC cases. The PanIN progression model indicates that early driver events may be suitable for molecular screening or liquid biopsy exploration in high-risk populations, whereas late tumor suppressor gene alterations may be more appropriate as indicators for progression risk assessment and prognostic evaluation.

Early KRAS-driven acinar-to-ductal metaplasia (ADM) represents an important intermediate state linking pancreatic tissue injury, chronic inflammation, and PanIN formation. ADM refers to the process by which mature acinar cells transform into a duct-like phenotype under conditions of injury, inflammation, or oncogenic KRAS activation. Physiological or transient ADM is to some extent reversible and contributes to tissue repair. However, when sustained KRAS activation, inflammatory stimulation, and epigenetic remodeling coexist, ADM may progress to PanIN and become a key early event in PDAC development (19). Chronic inflammation is not only a risk factor for PC but can also promote the stabilization of ADM, induce cellular plasticity, alter the stromal and immune microenvironment, and directly participate in the evolution of precancerous lesions.

The promoting effect of chronic pancreatitis on PanIN progression has been supported by relatively strong evidence from animal models and epidemiological studies. Experimental studies have shown that pancreatitis can significantly accelerate the progression from early PanIN to high-grade lesions and invasive carcinoma in mice harboring oncogenic KRAS. At the population level, chronic pancreatitis is also widely recognized as an important risk factor for PC (20). However, it should be noted that acute or chemically induced pancreatitis in animal models is not entirely equivalent to long-term chronic pancreatitis in humans. Differences in the duration of inflammation, microenvironmental composition, and genetic background may all affect the translational relevance of these findings.

At the level of clinical translation, the importance of preoperative histological sampling has become increasingly prominent. Endoscopic ultrasound-guided fine-needle biopsy (EUS-FNB) can be used not only to establish a pathological diagnosis but also to provide tissue samples for histological confirmation before neoadjuvant therapy, molecular testing, immune microenvironment assessment, and enrollment in clinical trials. Armellini et al. compared the performance of a 20G forward-cutting side-fenestrated biopsy needle with that of a 22G reverse-bevel side-fenestrated needle in EUS-guided sampling of solid pancreatic lesions. Their results suggested that the 20G side-fenestrated FNB needle achieved a higher rate of histological sample acquisition, providing technical support for preoperative histological evaluation and subsequent precision classification of PC (21). It should be emphasized that the value of EUS-FNB is not limited to positive diagnosis; it’s more important translational significance lies in obtaining sufficient tissue for the analysis of gene mutations, stromal composition, and immune phenotypes.

### 2.2 Smoking

Environmental factors play an indispensable role in the occurrence and progression of PC, including smoking, alcohol consumption, and a diet high in fat and protein (22). Epidemiological studies have indicated that smoking is positively correlated with the risk of PC, and exposure to secondhand smoke also increases the susceptibility to this disease (23). Exposure to cigarette smoke in the air stimulates the development of pancreatic intraepithelial neoplastic lesions. Cigarette smoke exerts a significant stimulatory effect on the number and size of pancreatic lesions, which are associated with the tumor microenvironment. Smoking reduces histone acetylation related to recruitment and phenotype in macrophages, further promoting the survival of precancerous and cancerous cells, leading to an eight-fold significant increase in pancreatic lesions (24).

A large number of harmful chemicals are generated during cigarette combustion (25), including nicotine, butadiene, aldehydes, bacterial endotoxins, free radicals, polycyclic aromatic hydrocarbons, tobacco-specific nitrosamines, and a high concentration of nitric oxide. These chemicals induce pancreatic inflammation and fibrosis, synergize with genetic factors, inhibit cell death, and stimulate cell proliferation, thereby promoting the occurrence and progression of PC. Inhalation of cigarette smoke may cause morphological damage to pancreatic exocrine glands, induce the gene expression of trypsinogen and chymotrypsinogen, further reduce pancreatic enzyme levels, and trigger pancreatic inflammation and fibrosis (26).

Nicotine activates the AKT-ERK-MYC signaling pathway, which leads to the inhibition of Gata6 promoter activity and the loss of GATA6 protein expression. Consequently, there is a loss of glandular differentiation, hyperactivation of oncogenic KRAS, enhanced tumor invasiveness, and induction of epithelial-mesenchymal transition (EMT), which increases the number of circulating tumor cells and their metastasis to the liver (27). Kumar and other researchers (28) demonstrated that tumor-associated macrophages (TAMs) primarily promote the upregulated expression of heparin-binding epidermal growth factor-like growth factor (HB-EGF) in preneoplastic lesions of mice exposed to cigarette smoke. Meanwhile, cigarette smoke exposure also causes partial immunosuppression in the early stages of PC progression.

It is therefore evident that the association between smoking and PC risk shows a high degree of epidemiological consistency and is supported by plausible mutagenic and pro-inflammatory mechanisms, making smoking a relatively well-established risk factor. However, a substantial proportion of the mechanistic evidence related to smoking still derives from animal or *in vitro* models. The interactions among different smoking doses, duration of smoking cessation, genetic background, and inflammatory status require further validation through more refined population-based studies. Its clearest clinical translational value remains primary prevention, namely smoking cessation and reduction of tobacco exposure.

### 2.3 Alcohol consumption

Studies have demonstrated that high alcohol intake is associated with an increased risk of PC, especially long-term heavy drinking, which can elevate the risk of PC by approximately 35% (29). The pancreas is a vital organ for the non-oxidative metabolism of alcohol. Ethanol and its metabolites (e.g., acetaldehyde) may affect the exocrine and endocrine functions of the pancreas. Approximately 70% of pancreatitis cases are caused by long-term heavy alcohol consumption, and chronic heavy drinking increases the risk of PC (30). Ethanol is metabolized by the cytochrome P450 system *in vivo*, generating large amounts of reactive oxygen species, which activate the NF-κB signaling pathway, promote the transcriptional activation of inflammatory factors, damage pancreatic tissue, and further accelerate the occurrence of PC (31).

Alcohol toxicity mainly depends on fatty acid ethyl esters produced from ethanol and fatty acids. Non-oxidative alcohol metabolites act directly on activated pancreatic stellate cells, induce cytoplasmic calcium overload, thereby disturbing mitochondrial function and triggering cell death, leading to pancreatic fibrosis and accelerating PC progression (32). In addition, ethanol can inhibit the activity of natural killer cells, activate monocytes and macrophages, and release tumor necrosis factor-α, interleukin-1, and interleukin-6. It also causes abnormalities in dendritic cells by impairing phagocytic function, exacerbating inflammatory responses, and reducing the ability of the immune system to recognize and eliminate abnormal cells, thus accelerating the development and immune escape of PC (33). However, evidence related to alcohol consumption is subject to relatively obvious confounding factors, particularly the combined effects of smoking, diet, chronic pancreatitis, and social behavioral factors. Compared with smoking, the strength of evidence supporting alcohol consumption as an independent risk factor is relatively weaker, and the association between low to moderate alcohol intake and PC risk remains controversial.

### 2.4 Obesity

Obesity is one of the important risk factors for PC. Epidemiological surveys have shown that more than 600 million people worldwide are affected by obesity (34). Since 1975, the global prevalence of obesity has tripled, which will further increase the global burden of PC (35). High body mass index (BMI) has been proven to increase the risk of PC. A study of more than 150,000 individuals (36) found that after adjusting for age, smoking, diabetes, and other risk factors, the risk of PC in people with BMI exceeding 30 kg/m² was 72% higher than that in people with BMI below 23 kg/m². Furthermore, not only is BMI change in adulthood associated with the risk of PC, but high BMI in childhood has also been shown to be associated with an increased risk of developing PC before the age of 70.

Adipokines act as mediators through which adipose tissue affects the whole body, and leptin and adiponectin are the two most important bioactive peptides (37). In the blood of PC patients with obesity, the level of leptin, which exerts pro-inflammatory effects, promotes tumor cell proliferation and angiogenesis, is increased, while the level of adiponectin, which possesses anti-inflammatory, anti-angiogenic and anti-tumor cell proliferation functions, is decreased (38). Leptin regulates glucose metabolism in PC cells and promotes tumor cell proliferation by activating the PI3K/Akt signaling pathway (39). Meanwhile, it can also activate the JAK2/STAT3 signaling pathway to upregulate the level of matrix metalloproteinase 13, promote extracellular matrix degradation, and accelerate lymph node metastasis of PC cells (40).

As a promising adipokine, Lipocalin-2 (LCN2) is significantly upregulated in the serum of obese PC patients, and its level is significantly lower after chemotherapy than before chemotherapy in human PC patients, suggesting that LCN2 may serve as a prognostic marker for PC (41). Gomez and other scholars (42) found that knockout of the Lcn2 gene in KRASG12D mice fed a high-fat diet reduced extracellular matrix deposition and immune cell infiltration, decreased the formation of pancreatic intraepithelial neoplasia, and delayed PC progression, whereas downregulation of the LCN2-specific receptor SLC22A17 abolished these effects. Obesity-related mechanisms have a solid epidemiological basis, but most of their downstream molecular pathways are derived from preclinical studies and have not yet led to mature interventional targets. In particular, although biomarkers such as LCN2, leptin, and adiponectin are associated with PC prognosis and metabolic status, large-scale prospective validation is still lacking to determine whether they can serve as stable early diagnostic indicators or therapeutic targets.

### 2.5 Genetic Factors

PC is a complex disease caused by the interaction between genetic and environmental factors. The occurrence of tumors is closely related to the individual genetic background, indicating that genetic factors play an important role in the pathological progression of PC. Compared with environmental factors, the genetic component has stronger mechanistic certainty and greater clinical translational value, particularly in high-risk population screening, genetic counseling, molecular classification, and targeted therapy.

#### 2.5.1 Genomic mutations in PC

The occurrence and progression of PC are accompanied by a large number of gene mutations. Genes closely associated with the pathogenesis of PC include TP53, SMAD4, CDKN2A, ARID1A and ROBO2, as well as novel candidate driver genes of PC such as KDM6A and PREX2 (43). Relevant genomic studies have revealed substantial heterogeneity in PC-mutated genes, and this diversity also explains why traditional clinical research designs fail to demonstrate efficacy in other non-enrolled PC patients (44). Genomic stability is disrupted in the majority of cancer patients, and PC patients are no exception. Approximately 97% of PC patients exhibit genomic rearrangements, manifested as gene locus mutations, amplifications, deletions, translocations and inversions (45).

Approximately 90% of PC patients harbor KRAS mutations, which are mainly concentrated in three codons, namely G12, G13 and Q61. This is also one of the most common oncogenic events in PC patients. As the most important triggering factor for PC initiation, KRAS mutation also signifies the transformation from normal cells to initiated cells (46). As a small GTPase, KRAS can activate the MAPK-ERK signaling pathway, thereby regulating multiple cellular biological processes. Its mutation has been proven to enhance cellular adaptability to the microenvironment and promote the survival and proliferation of tumor cells by altering metabolic pathways, resisting inflammation-related senescence, affecting autophagy, and upregulating stress granules (47).

CDKN2A is located on chromosome 9 and encodes proteins such as p16 and p14. As a key tumor suppressor gene, it can inhibit cell cycle progression by binding to and suppressing cyclin-dependent kinases 4 and 6 (CDK4/6), thereby exerting its tumor-suppressive effects. Studies have shown that germline mutations in the p16 tumor suppressor gene can induce PC, whereas whether germline mutations in p14 exist remains unclear (48). The p16 protein can inhibit the activity of CDK4 and CDK6, thereby preventing the inactivating phosphorylation of the RB protein, ultimately leading to cellular senescence and cell cycle arrest. Pancreatic ductal adenocarcinoma (PDAC) with CDKN2A inactivation exhibits sensitivity to certain drugs (e.g., paclitaxel) (49), suggesting potential therapeutic strategies for tumors harboring these mutations. The concurrent occurrence of PC and melanoma in the same patient indicates hereditary susceptibility, which in some cases is caused by germline CDKN2A mutations (50).

Unlike the two aforementioned genes, the inactivation of TP53 and SMAD4 are relatively late events in PC development. Their occurrence indicates the presence of potentially lethal tumors and is considered to be associated with aggressive PC (18). The TP53 gene is located on chromosome 17 and exerts its tumor-suppressive effect by regulating cell division. It is weakly expressed in normal cells but highly expressed in malignant tumors. TP53 mutations are detected in 50–90% of PC patients and often co-occur with KRAS mutations, which is indicative of early-stage PC onset (51). Mutant P53 accumulates in PC patients, loses its normal functions, and is unable to induce the expression of transcriptionally active genes such as P21. Among these, the P53 TAD2 mutant can act as a super tumor suppressor gene in PC by hyperactivating Ptpn14 to negatively regulate Yap. This study demonstrates that the p53-Ptpn14-Yap axis serves as a key tumor-suppressive axis in PC and may be exploited as a potential therapeutic target for PC (52). Meanwhile, TP53 mutations significantly affect the tumor microenvironment of PC, modulating immune responses, T-cell differentiation, and the interactions of cancer-associated fibroblasts.

SMAD4 mediates the proliferation and apoptosis of pancreatic cells. Mutations or deletions of SMAD4 are detected in more than 20% of patients with pancreatic ductal adenocarcinoma (PDAC) (53), and SMAD4 is specifically inactivated in 50% of advanced PC cases (54). SMAD4 encodes proteins involved in the Transforming growth factor-β (TGF-β) and Bone morphogenetic protein (BMP) signaling pathways. TGF-β is a pleiotropic protein with both pro-tumorigenic and anti-tumorigenic effects, and has been identified as a potent inducer of EMT. This process endows epithelial cells with migratory and invasive properties during PC progression, thus aberrant TGF-β signaling and EMT are closely associated with the aggressiveness of PC (55). The TGF-β-SMAD4 pathway also exerts anti-tumor effects by promoting the transcription of cell cycle inhibitors (e.g., p15, p21 and p27). These inhibitors can block cyclin-dependent kinases and induce growth arrest and apoptosis by upregulating the transcription of pro-apoptotic genes (56). When SMAD4 loses its normal function due to missense, nonsense or frameshift mutations, the canonical TGF-β-SMAD4 signaling axis is abrogated, thereby impairing its tumor-suppressive activity.

In addition to the aforementioned gene mutations, other identified mutated genes include MLL3, TGFBR2, ARID1A and SF3B1, as well as EPC1 and ARID2 which are involved in chromatin modification, ATM which participates in DNA damage repair, and novel mutated genes such as ZIM2, MAP2K4, NALCN, SLC16A4 and MAGEA6 that are associated with other mechanisms (57).

#### 2.5.2 Susceptibility gene mutations in PC

With the development of high-throughput sequencing technology, genome-wide association study (GWAS) has become a major strategy for identifying susceptibility-related genes and genetic loci of malignant tumors. Wolpin et al. (58) conducted a multi-stage GWAS involving 7,683 PC patients and 14,397 controls of European ancestry. Four novel loci (7q32, 16q23.1, 13q12.2 and 22q12.1) reached genome-wide significance, and two additional susceptibility loci (5p15.33 and 8q24.2) were identified. Petersen and other scholars (59) performed a GWAS on 3,851 PC patients and 3,934 unaffected controls, which confirmed three common susceptibility loci of PC located on chromosomes 13q22.1, 1q32.1 and 5p15.33, respectively.

However, the situation differs in Asian populations. PC ranks as the fifth leading cause of cancer-related deaths in Japan. Low and other researchers (60) conducted a GWAS involving 991 cases of invasive pancreatic ductal adenocarcinoma and 5,209 controls, and identified three genomic loci on chromosomes 6p25.3, 12p11.21 and 7q36.2 that are significantly associated with PC susceptibility. Wu and other researchers (61) performed a GWAS on 2,603 PC patients and 2,877 controls recruited from 25 hospitals across 16 provinces and municipalities in China. The study identified five novel susceptibility loci on chromosomes 21q21.3, 5p13.1, 21q22.3, 22q13.3 and 10q26.11, which may also serve as potential targets for PC prevention or treatment.

It should be noted that most loci identified by GWAS are located in noncoding regions, and their functions often do not directly alter protein structure. Instead, they influence disease risk by regulating gene expression, chromatin accessibility, enhancer activity, or lncRNA function (62). Although mechanistic studies have been conducted for some loci, most susceptibility loci still lack clear biological explanations. Therefore, at the current stage, the value of genetic susceptibility research lies mainly in risk stratification and the discovery of mechanistic clues, rather than in directly guiding treatment. Future studies should integrate GWAS, whole-exome sequencing, single-cell omics, organoid models, and clinical phenotypic data to improve the interpretability and clinical applicability of genetic findings.

### 2.6 Immune factors

PC is characterized by a highly heterogeneous tumor microenvironment (TME). The TME is a complex system composed of tumor cells, immune cells, cancer-associated fibroblasts (CAFs), extracellular matrix, cytokines, and other molecules that contribute to tumor growth and progression, which surround and support the tumor nest (63). The unique TME of PC is characterized by low infiltration of cytotoxic T cells but abundant immunosuppressive cells, resulting in poor efficacy of conventional immunotherapy against PC. During PC progression, PC cells exhibit significantly enhanced metabolic adaptability and glycolysis, leading to the accumulation of lactate and other metabolites in the tumor microenvironment. This in turn recruits immunosuppressive cells such as myeloid-derived suppressor cells and tumor-associated macrophages, forming a microenvironment that promotes tumor proliferation, metastasis, and resistance to immune attack (64). Excessively accumulated lactate in the immune microenvironment drives histone H3K18 lactylation modification and promotes cholesterol synthesis and release. Accumulated cholesterol polarizes tumor-associated macrophages toward the M2 phenotype and inhibits the activity of CD8^+^ T cells (65).

Lactate metabolism interacts with multiple signaling and metabolic pathways in PC. It not only serves as a substrate for PC energy metabolism but also directly promotes tumor growth and invasion through multiple mechanisms. Studies have shown that lactate produced by hypoxic tumor cells can be taken up by adjacent cancer cells or fibroblasts in normoxic regions, converted into pyruvate, and oxidized in mitochondria, thereby providing an alternative energy source under glucose deprivation (66). Lactate also activates transcription factors such as HIF-1α and NF-κB. In the high-lactate and hypoxic environment, oncogenic factors including HIF-1α and c-Myc are persistently activated, driving the sustained activity of downstream signaling pathways such as vascular endothelial growth factor (VEGF) and its receptor VEGFR2, as well as NF-κB. This not only promotes tumor angiogenesis to support tumor growth but also induces the expression of pro-proliferative genes, forming a positive feedback loop for tumor progression (67).

A large number of regulatory T cells exist in the PC microenvironment. They can inhibit the proliferation and activation of effector T cells by expressing cytotoxic T lymphocyte-associated protein 4 and secreting IL-35. Regulatory T cells also suppress the antigen-presenting function of dendritic cells and block the immune response (68). Among these factors, lactate dehydrogenase (LDH) is a key enzyme in the glycolytic pathway, and serum LDH levels reflect the systemic lactate metabolism of tumors. A retrospective analysis of patients with advanced PC showed that patients with low serum LDH levels had significantly longer survival, indicating that serum LDH can serve as an important biomarker for evaluating the invasiveness and prognosis of PC (69). Lactate dehydrogenase A (LDHA) is the preferred target for regulating lactate production. In mouse models of PC, FX11, as an LDH inhibitor, reduces intratumoral lactate levels, delays tumor progression, and enhances anti-tumor immune responses, including increased infiltration of CD8^+^ T cells and NK cells (70).

CAF heterogeneity is a key issue in explaining immune exclusion and therapeutic resistance in PC. Early studies often regarded CAFs as a single tumor-promoting cell population. However, single-cell sequencing and spatial analyses have shown that CAFs comprise at least several subtypes, including myofibroblastic CAFs (myCAFs), inflammatory CAFs (iCAFs), and antigen-presenting CAFs (apCAFs) (71, 72). myCAFs are typically located adjacent to tumor cells and express stromal-associated molecules such as α-SMA and COL1A1, participating in ECM deposition, increased tissue stiffness, and the formation of barriers to drug delivery. iCAFs are mainly distributed in stromal regions relatively distant from cancer cells and secrete inflammatory mediators such as IL-6, LIF, and CXCL1, thereby promoting myeloid cell recruitment and immunosuppression. apCAFs express antigen presentation-related molecules such as MHC-II and CD74 but lack classical costimulatory molecules, which may induce CD4+ T-cell tolerance or Treg formation.

The mechanism of immune exclusion is not caused simply by “excessive stroma,” but rather by the combined effects of CAFs, ECM, chemokines, and immune checkpoints. FAP+ CAFs can secrete CXCL12 and confine T cells outside tumor nests, while blockade of the CXCL12/CXCR4 axis has been shown to enhance the response to anti-PD-L1 therapy in animal models (73). Meanwhile, human-derived CAFs can induce the expression of inhibitory receptors, including PD-1, TIM-3, CTLA-4, and LAG-3, on CD4+ and CD8+ T cells and impair T-cell cytotoxic function (74). Notably, however, direct depletion of α-SMA+ CAFs or broad attenuation of the fibrotic stroma may instead enhance immunosuppression and accelerate tumor progression in some models (75). This suggests that CAFs are not a simple therapeutic target but rather a dynamic cell population with both tumor-promoting and tumor-suppressive functions. Future therapeutic strategies should avoid nonselective “stromal depletion” and instead pursue precise modulation based on CAF subtypes, spatial distribution, and immune status.

## 3 Diabetes mellitus and PC

Epidemiological studies have confirmed that diabetes mellitus is an important risk factor for PC. Patients with diabetes mellitus have a 1.5–2.0-fold increased risk of developing PC, especially those with long-standing type 2 diabetes mellitus (76). Long-term diabetes is considered a crucial risk factor for PC development, and the use of certain antidiabetic drugs can modulate the risk of PC. Meanwhile, metabolic disorders represented by hyperglycemia and hyperinsulinemia, insulin resistance, and systemic chronic inflammatory responses may all promote the occurrence and progression of PC (13).

Hyperglycemia is one of the key characteristics of type 2 diabetes mellitus. Sustained exposure to hyperglycemia as well as prediabetic status can significantly increase the risk of PC (77). Diabetes mellitus is closely associated with PC; approximately 85% of PC patients develop glucose intolerance or even impaired glucose metabolism. Hyperglycemia may promote the invasiveness and migration ability of PC cells by generating hydrogen peroxide to regulate the expression of EMT-related factors in PC tissues (78). Blood glucose provides energy for cancer cells, thereby facilitating the growth of malignant cells. Patients with poorly controlled glycometabolism are often accompanied by elevated levels of chronic inflammatory markers. Uncontrolled pro-inflammatory responses induce a state of chronic inflammation, which forms a tumor-promoting microenvironment and leads to excessive immune activation and cancer progression (79). Notably, PDAC exhibits high chemoresistance, while hyperglycemic status significantly enhances the efficacy of conventional single-agent and multi-agent chemotherapy regimens against PDAC (80).

Hyperglycemia can promote the occurrence and progression of PC through multiple pathways. Eighty-five percent of PC patients are accompanied by impaired glucose tolerance or even diabetes mellitus. Under hyperglycemic conditions, the activities of lactate dehydrogenase A (LDHA) and the expression levels of hexokinase 2 (HK2), phosphofructokinase platelet (PFKP) are significantly upregulated. Hypoxia-inducible factor-1α (HIF-1α) accumulation induced by hyperglycemia can increase LDHA activity and the expression of HK2 and PFKP, thereby enhancing glycolysis in PC to facilitate cancer progression (81). Hyperglycemia may promote immune escape of PC cells in a hyperglycemic tumor microenvironment. Modulating the cell surface expression of major histocompatibility complex class I chain-related proteins A and B (MICA/B) has been proven to be one of the mechanisms by which tumor cells evade natural killer (NK) cell-mediated killing. Hyperglycemia protects PC cells from NK cell-mediated killing by inhibiting the expression of MICA/B (82).

Molecules associated with the Wnt/β-catenin signaling pathway are overexpressed in PC tissues/cells and are closely correlated with poor prognosis in PC patients. Hyperglycemia exacerbates the aberrant activation of β-catenin in PC and enhances the malignant biological behaviors of PC in a Wnt/β-catenin signaling pathway-dependent manner (83). Kato and other researchers (84) demonstrated that metformin, a commonly used oral hypoglycemic agent, can inhibit the proliferation of PC cell lines Panc1, PK1 and PK9 *in vitro*. It also reduces the phosphorylation of epidermal growth factor receptor (EGFR) at Tyr845 and the expression of insulin-like growth factor 1 receptor (IGF-1R) both *in vitro* and *in vivo*. Metformin may inhibit PC cell cycle-related molecules by altering miRNAs, exhibiting potential to reduce PC risk and improve prognosis.

However, whether antidiabetic medications can significantly reduce the incidence of PC or improve prognosis remains heterogeneous across studies and is substantially influenced by indication bias, duration of diabetes, tumor stage, and treatment regimens. Therefore, the clinical translational focus of diabetes-related mechanisms should be placed on the identification of high-risk populations, biomarker screening for new-onset diabetes, and evaluation of the impact of metabolic status on treatment response.

## 4 Pancreatitis and PC

Pancreatitis and PC represent two major disorders of the exocrine pancreas. Pancreatitis manifests in two forms: acute and chronic. Recurrent episodes of acute pancreatitis activate pancreatic stellate cells, induce irreversible damage to the pancreatic gland, and accelerate the progression of pancreatic atrophy and fibrosis in chronic pancreatitis (85). In recent years, a growing body of evidence has identified long-standing chronic pancreatitis as a strong risk factor for PC development. Post-pancreatitis diabetes mellitus and excessive intrapancreatic fat deposition can specifically affect the tumor macroenvironment and microenvironment within the pancreas (86).

The transformation of chronic pancreatitis to PC is a complex and prolonged process. Kudo and other researchers (87) conducted a follow-up study on 218 patients with chronic pancreatitis, among whom 9 progressed to PC. The average time from the diagnosis of chronic pancreatitis to that of PC was 9.6 years, and approximately 5% of patients with chronic pancreatitis will develop PC (88). Acinar cells of the digestive glands that secrete enzymes may undergo acinar-ductal metaplasia in the inflammatory microenvironment of pancreatitis. This metaplastic change is recognized as a precursor of PC. Meanwhile, oxidative stress and inflammatory responses can promote the progression of pancreatitis and interact with genetic factors (such as oncogenic KRAS mutations and inactivation of tumor suppressor genes), thereby initiating and accelerating pancreatic intraepithelial neoplasia (89). Inflammatory molecules can also promote tumor growth through autocrine and paracrine effects of epithelial and stromal cells (90).

Alterations in intrapancreatic neural structures are a typical feature coexisting in patients with chronic pancreatitis and PC. The size and quantity of intrapancreatic nerves increase significantly, the ratio of autonomic nerve fibers to sensory fibers is reversed, and these nerves are infiltrated by perineural inflammatory cells or invaded by PC cells (91). Lin and other researchers (92) demonstrated that enhanced IL-22 signaling induced by chronic pancreatic inflammation promotes KRAS mutation-mediated STAT5 phosphorylation. STAT5 activation is a key effector downstream of oncogenic KRAS signaling, and is crucial for acinar-to-ductal metaplasia and PC progression. Overall, the evidence chain linking chronic pancreatitis to PC is relatively strong. However, the clinical challenge lies in identifying patients with chronic pancreatitis who are truly at high risk of progressing to PC and in establishing safe and sustainable surveillance strategies.

## 5 Pathway mechanisms

### 5.1 PI3K/Akt/mTOR signaling pathway

Aberrant activation of the PI3K/Akt/mTOR pathway plays a crucial role in tumorigenesis and is closely associated with multiple tumorigenic processes including cell proliferation, autophagy, apoptosis, angiogenesis, EMT and chemoresistance (93). In the PI3K/AKT pathway, PI3K can upregulate the expression of matrix metalloproteinases (MMPs), among which MMP-2 and MMP-9 exert prominent effects on promoting the metastasis and progression of PC cells (94). p130Cas is an adaptor protein that mediates signal transduction of both integrins and growth factor receptors. As an important downstream effector of KRAS, it promotes PI3K activation and induces acinar-ductal metaplasia of pancreatic acini, which further progresses to tumors (95). Upon stimulation by corresponding receptors, PI3K regulates phosphatidylinositol-3,4,5-trisphosphate (PIP3) to induce the phosphorylation and activation of Akt kinase (Akt). Activated Akt can regulate various downstream proteins such as mammalian target of rapamycin (mTOR), thereby modulating cellular processes including autophagy and apoptosis (96).

**Figure 2:**
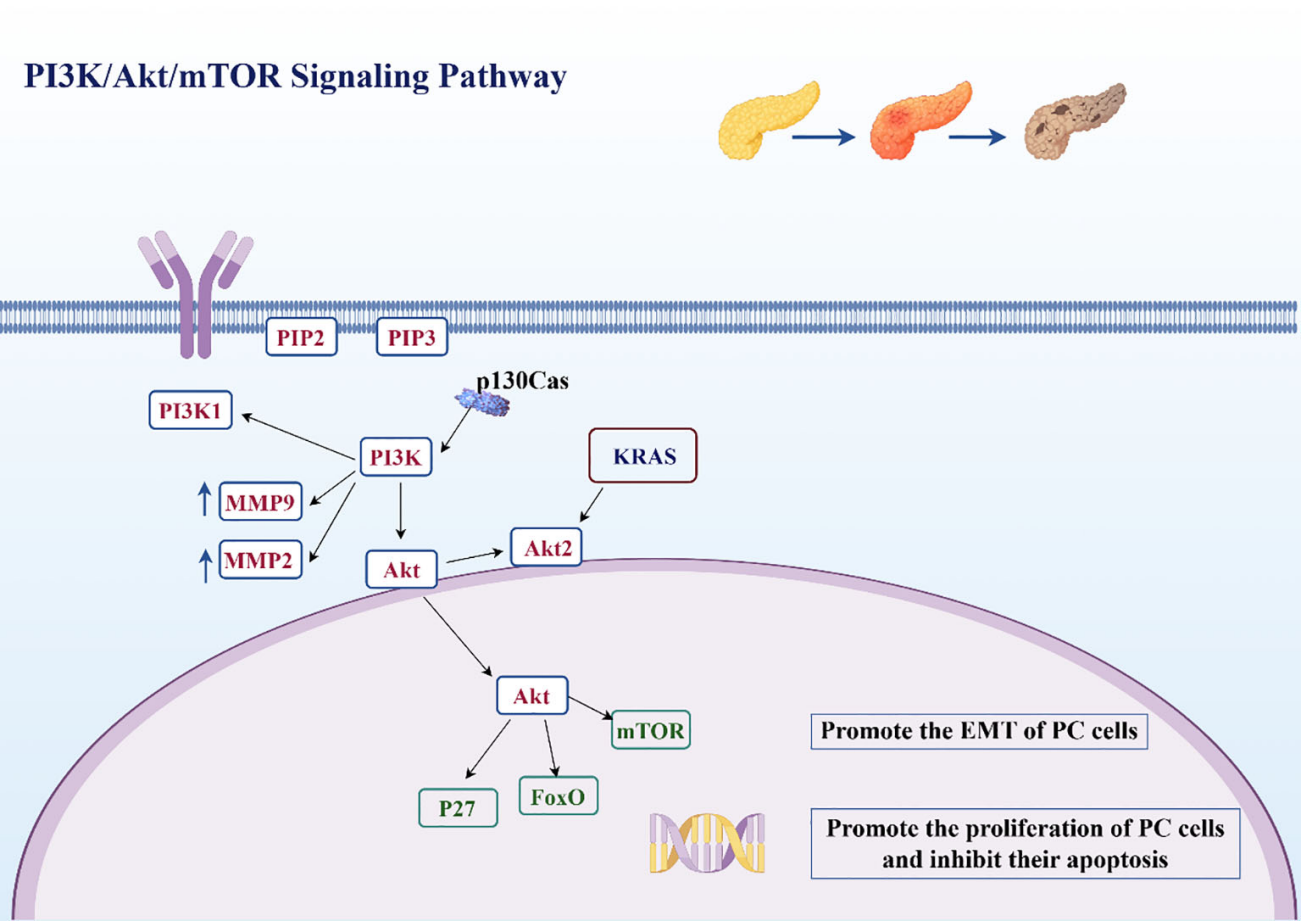
The role of the PI3K/Akt/mTOR signaling pathway in the pathogenesis of pancreatic cancer.

AKT activation consists of two phosphorylation processes and includes three subtypes. Among these, AKT2 is expressed in islet effector cells. Long-term stimulation by a high-fat diet triggers Akt2, leading to significant proliferation of islet cells and thus disrupting the balance between islet cell proliferation and apoptosis (97). Activated AKT exerts multiple biological functions, including activating the cAMP response element-binding protein, translocating the forkhead box transcription factor FoxO, inhibiting the cyclin-dependent kinase inhibitor p27, and activating the mTOR. These effects induce the growth and apoptosis of PC cells and promote protein synthesis (98) (**Figure 2**). From the perspective of evidence strength, the PI3K/Akt/mTOR pathway is a mechanistically well-defined pathway in PC with abundant preclinical evidence. However, the clinical efficacy of simply inhibiting this pathway is often limited by feedback activation, pathway redundancy, and toxicity. Future studies should place greater emphasis on the stratified value of this pathway in the context of KRAS status, metabolic conditions, and combination treatment strategies.

### 5.2 Wnt/β-catenin signaling pathway

The Wnt signaling pathway is one of the crucial signaling pathways involved in regulating stem cell differentiation, organogenesis and cell survival, and serves as a potential regulatory target for various tumors (99). The Wnt gene was first identified in Drosophila melanogaster as a mutation that causes winglessness. Subsequently, a conserved locus was discovered on chromosome 15 in mice, which was designated as int. The Drosophila gene and the mouse gene were found to be homologous, hence the combined name Wnt was adopted (100).

In the canonical pathway, when the Wnt receptor is not bound by a ligand, β-catenin is phosphorylated by the destruction complex and then degraded. When the ligand binds to the receptor, it induces the phosphorylation and activation of Dishevelled protein, and dissociates glycogen synthase kinase-3β (GSK-3β) from the destruction complex. After GSK-3β dissociates from the destruction complex, β-catenin cannot be phosphorylated and degraded. Instead, it accumulates in large quantities in the cytoplasm, translocates into the nucleus, and regulates the expression of target genes (101).

**Figure 3:**
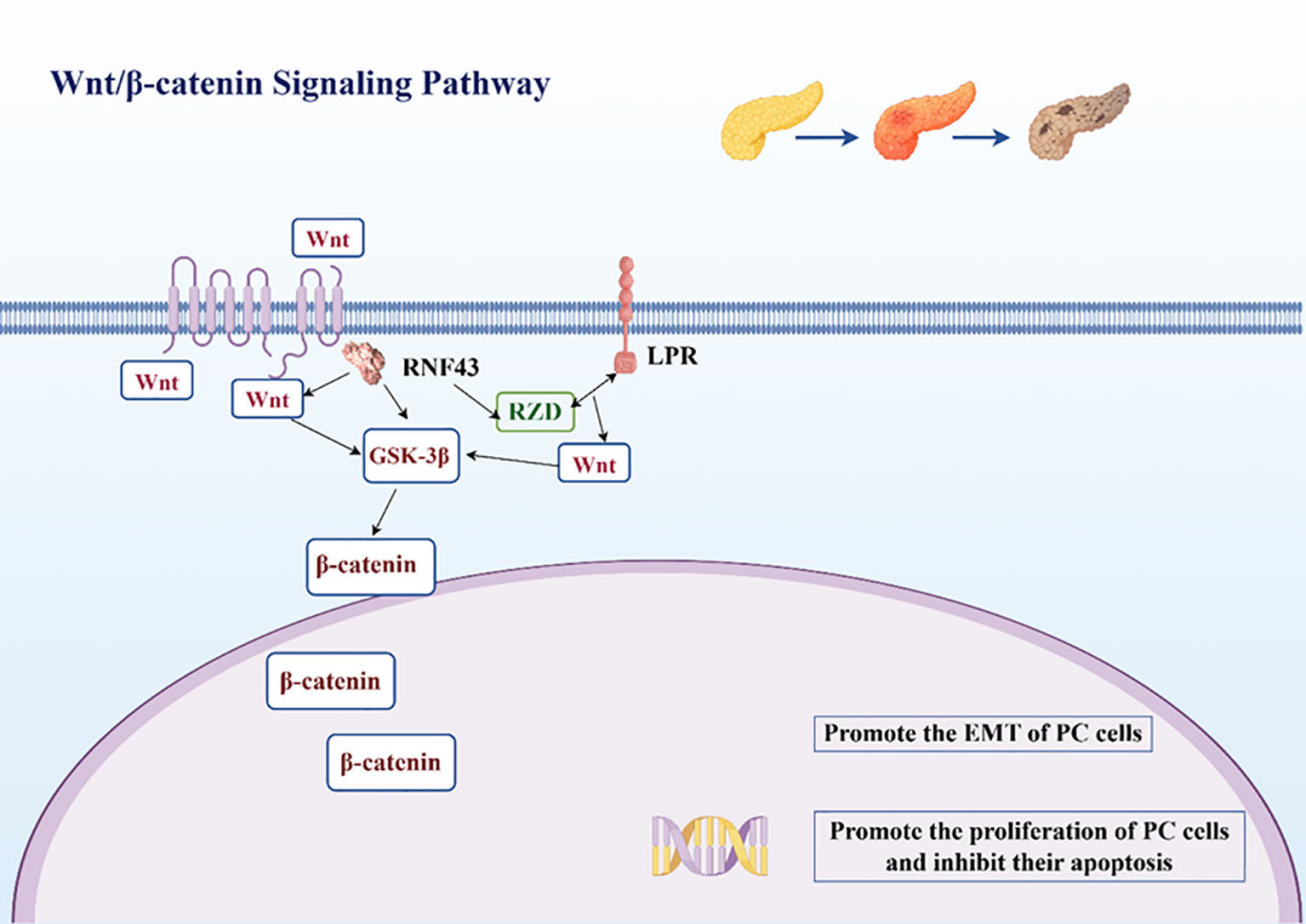
The role of the Wnt/β-catenin signaling pathway in the pathogenesis of pancreatic cancer.

Pancreatic cystic lesions (PCLs) are precursor lesions of PC. Intraductal papillary mucinous neoplasms (IPMNs) represent a major type of PCLs. Ring finger protein 43 (RNF43), an E3 ubiquitin ligase, is also the most frequently mutated gene in IPMNs. It can mediate the ubiquitination and degradation of the Wnt ligand-frizzled complex, and inhibit its transcriptional activity by anchoring transcription factor 4 (TCF4) to the nuclear membrane, thereby activating the Wnt/β-catenin signaling pathway (102) (**Figure 3**). The role of the Wnt/β-catenin pathway in IPMN and certain subsets of PC has strong biological plausibility, but marked differences exist among different precursor lesions and histological subtypes. Therefore, the Wnt pathway is more appropriately considered as part of the risk assessment for progression in specific molecular subtypes or cystic lesions, rather than being generalized as a universal driving mechanism across all PC.

### 5.3 NF-κB signaling pathway

The NF-κB signaling pathway plays an important role in regulating immune and inflammatory responses. It is involved in modulating the expression of various genes, exerts an extremely crucial effect on cellular processes including proliferation, differentiation, survival and apoptosis, and is associated with the expression of angiogenic factors, adhesion molecules and oncogenes related to growth regulation during tumorigenesis and progression (103). The NF-κB protein family comprises RelA (p65), RelB, c-Rel, p50 (precursor of p105) and p52 (precursor of p100), and is composed of ligands, receptors, receptor-proximal signaling adaptor proteins, inhibitor of NF-κB (IκB) and NF-κB dimers (104).

In the canonical pathway, the NF-κB complex consists of two subunits, p65 and p50. Ligand binding to their respective receptors (such as Toll-like receptors, TNF receptors, and IL-1 receptors) induces conformational changes of the receptors, which in turn activates the inhibitor of NF-κB kinase (IKK) proteins. This releases NF-κB from the cytoplasmic dimer, which then translocates into the nucleus to regulate the expression of genes involved in relevant biological processes. In the non-canonical pathway, receptors of different classes trigger kinase activation to induce NF-κB phosphorylation, and mainly activate IKK1 to mediate the phosphorylation of p100 into p52; the resulting complex composed of RelB and p52 then exerts its biological functions (105).

Activation of NF-κB signaling can promote the progression of PC. Interleukin-1 receptor-associated kinase 2 (IRAK2), which is overexpressed in PC specimens, can drive glycolysis in PC cells by enhancing the phosphorylation of NF-κB, providing bioenergy for the survival and proliferation of PC cells and accelerating PC growth (106). Other target genes of NF-κB, such as vascular endothelial growth factor (VEGF), fibroblast growth factor and platelet-derived growth factor, can cooperate with IL-8 to mediate vascular basement membrane degradation and extracellular matrix (ECM) remodeling, thereby promoting angiogenesis (107). Chen and other researchers (108) demonstrated that knockdown of AHNAK2 leads to inhibited tumor growth and prolonged survival time in PDAC mice, and can significantly suppress the proliferation, migration and invasion of PDAC cells. The AHNAK2 gene belongs to the AHNAK protein family; it is highly expressed in PC tissues and correlated with poor prognosis, thus may serve as a potential prognostic biomarker for PDAC patients (109).

**Figure 4:**
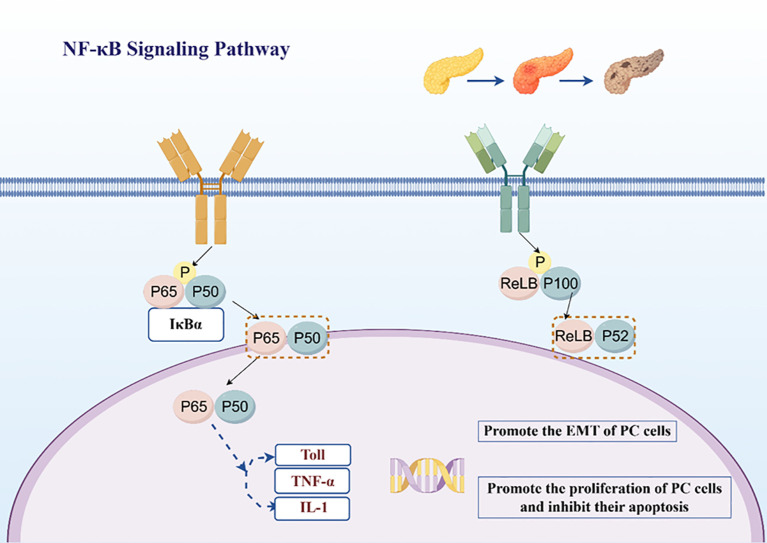
The role of the NF-κB signaling pathway in the pathogenesis of pancreatic cancer.

Mechanistically, activation of NF-κB reverses the knockdown of the AHNAK2 gene and reduces the expression of phosphorylated p65, phosphorylated IκBα, and matrix metalloproteinase-9 (MMP-9), which is associated with the inhibition of NF-κB/MMP-9 signaling pathway activation (110). Macrophages can activate NF-κB by upregulating the expression of TNF receptor-associated factor 6 in PC cells via CC motif chemokine ligand 5, enhance the non-autonomous activation of TNF-like weak inducer of apoptosis in PC cells, thereby promoting denervation-induced muscle atrophy and contributing to the development of cachexia in PC patients (111) (**Figure 4**). The advantage of the NF-κB pathway lies in its ability to link chronic inflammation, metabolic reprogramming, and immunosuppression, making it one of the important mechanisms explaining the transition from pancreatitis to PC. However, because NF-κB is broadly involved in normal immune responses, direct systemic inhibition is associated with toxicity and insufficient specificity. Therefore, its translational value is more likely to be reflected in targeting upstream inflammatory nodes, specific cellular populations, or combination therapeutic strategies.

### 5.4 TGF-β/Smads signaling pathway

TGF-β can stimulate the activation and proliferation of fibroblasts, thereby leading to extracellular matrix deposition. It exerts multiple homeostatic functions in inflammation regulation, cell proliferation, differentiation and the healing of various tissues, and its expression level is usually closely correlated with disease severity (112). There are three mammalian isoforms of the TGF-β gene, namely TGF-β1, TGF-β2 and TGF-β3, among which TGF-β1 has the closest association with cancer. TGF-β has three types of receptors (TGF-βRI, TGF-βRII and TGF-βRIII). Smad proteins act as mediators between ligands and receptors, and are known to be classified into nine categories. Among these, the phosphorylation of Smad3 by TGF-βRII is a key step in signal transduction (113, 114). TGF-β first binds to TGF-βRII and triggers the phosphorylation of TGF-βRI. The activated TGF-βRI/TGF-βRII complex further phosphorylates Smad2 and Smad3 proteins. These two proteins bind to Smad4 to form a complex, and the activated complex can interact with other transcription factors to regulate TGF-β signaling (115, 116).

TAMs are an important component of the tumor microenvironment and participate in processes such as angiogenesis, growth and metastasis of PC. TGF-β1 is produced by all leukocyte lineages, including lymphocytes, macrophages and dendritic cells, and its expression coordinately regulates the differentiation, proliferation and activation status of these immune cells through autocrine and paracrine modes (117). Zhou and other researchers (118) demonstrated through their research that TGF-β1 is positively correlated with the growth of human PC. Disruption of TGF-β1 can inhibit PC growth stimulation, significantly enhance anti-tumor immunity, and sensitize PC to chemotherapy. Targeting TGF-β1 may thus emerge as a breakthrough for the treatment of highly lethal PC.

**Figure 5:**
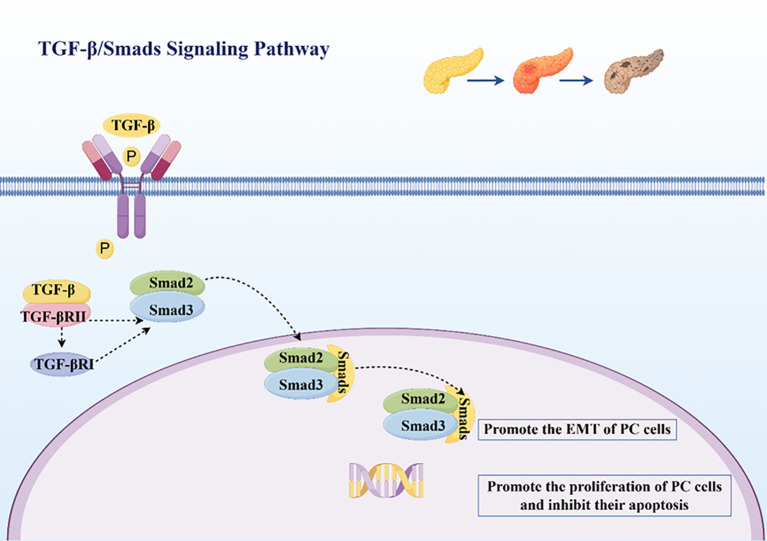
The role of the TGF-β/Smads signaling pathway in the pathogenesis of pancreatic cancer.

In the early stages of PC, the TGF-β signaling pathway delays the growth and migration of PC by inhibiting cell proliferation and inducing apoptosis. In the advanced stages of PC, however, cancer cells evade the inhibitory effects of TGF-β and instead exploit this pathway to promote tumor angiogenesis, cancer cell division and differentiation, thereby accelerating the progression of PC (119, 120) (**Figure 5**). The evidence regarding the TGF-β pathway is relatively complex, involving both strong mechanistic support and marked context dependence. Therefore, this pathway should not be simply classified as either a “tumor-promoting pathway” or a “tumor-suppressive pathway.” Future studies should incorporate stratification based on SMAD4 status, CAF subtypes, tumor stage, and immune infiltration characteristics to avoid unintended consequences resulting from nonselective inhibition.

### 5.5 JAK2/STAT3 signaling pathway

Janus kinase (JAK)/signal transducer and activator of transcription (STAT) is a transduction pathway activated by cytokines, which regulates cellular processes including survival, proliferation and tumorigenesis. Among these components, STAT3 has been extensively studied in the fields of inflammation and cancer research. Upon stimulation by upstream signals, JAK2 is activated through receptor tyrosine phosphorylation, which in turn induces the phosphorylation of STAT3 (121). The STAT protein then dissociates from the receptor, translocates to the nucleus, binds to corresponding target sites and modulates the transcription of downstream genes, thereby regulating various cellular processes.

**Figure 6:**
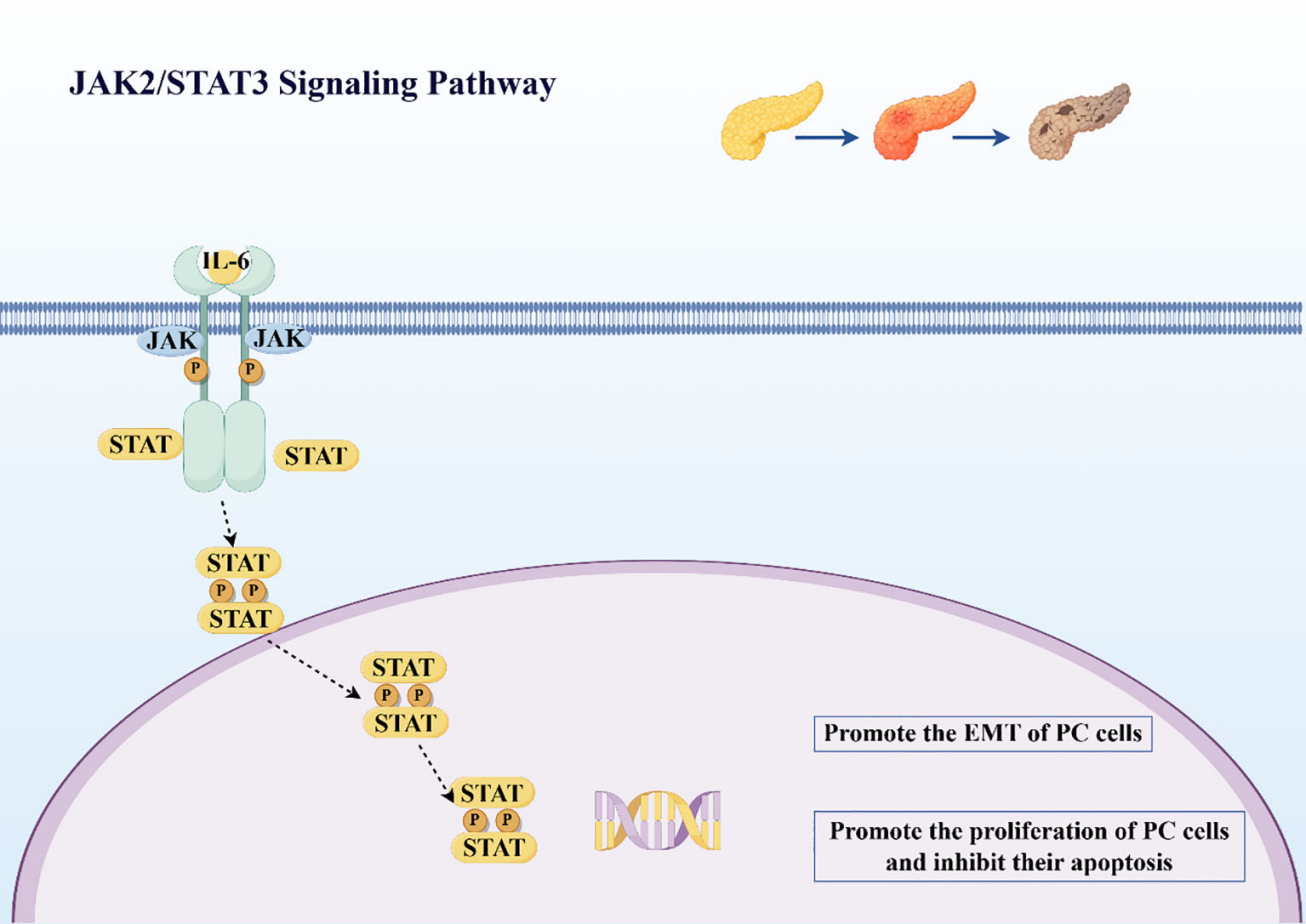
The role of the JAK2/STAT3 signaling pathway in the pathogenesis of pancreatic cancer.

Interleukins (ILs) are a group of cytokines that regulate immune responses in the body, including pro-inflammatory cytokines represented by IL-1β, IL-6 and IL-17, as well as anti-inflammatory cytokines represented by IL-10 (122). Among these, IL-6 can induce pancreatic intraepithelial neoplasia and mediate the EMT process, leading to tumor cell migration and invasion (123). Activation of the JAK2/STAT3 signaling pathway stimulates the production of more fibroblast growth factors in the tumor microenvironment of PC, thereby exacerbating disease progression, inhibiting cancer cell recognition and immune responses, and promoting the IL-6-triggered immune escape process (124) (**Figure 6**). The advantage of the JAK2/STAT3 pathway lies in its ability to link inflammation, CAF activation, immunosuppression, and therapeutic resistance. However, the clinical benefits of its inhibitors remain inconsistent, and STAT3 is broadly involved in maintaining normal tissue homeostasis. Therefore, greater emphasis should be placed on exploring this pathway as a synergistic target for combination immunotherapy or anti-stromal therapy, rather than as a standalone therapeutic breakthrough.

### 5.6 p53 signaling pathway

The p53 protein is a critically important tumor suppressor gene product in humans that is involved in regulating a variety of biological processes in human cells. Mutations or deletions in this pathway are closely associated with the development of PC. Meanwhile, p53 mutations are recognized as the second most common type of mutation in PC, leading to the formation of neomorphic mutant proteins. These mutants selectively regulate distinct metabolic pathways in PC cells and significantly promote PC metastasis. p53^R270H^ induces alterations in the expression of genes associated with oxidative stress and reduced mitochondrial respiration. In contrast, p53^R172H^ specifically affects the expression levels of enzymes related to urea metabolism (125). In the absence of relevant stress stimuli, P53 is catalyzed to undergo ubiquitination by E3 ligase, and then degraded via the proteasomal pathway, thus being unable to exert its biological functions. Upon exposure to stress, P53 is phosphorylated by checkpoint kinase 1 (Chk1) and checkpoint kinase 2 (Chk2). Chk1 and Chk2 are activated by ataxia-telangiectasia and Rad3-related gene (ATR) and ataxia-telangiectasia mutated gene (ATM), respectively. These kinases subsequently phosphorylate P53, and the activated P53 binds to chromatin to regulate the expression of target genes (126).

**Figure 7:**
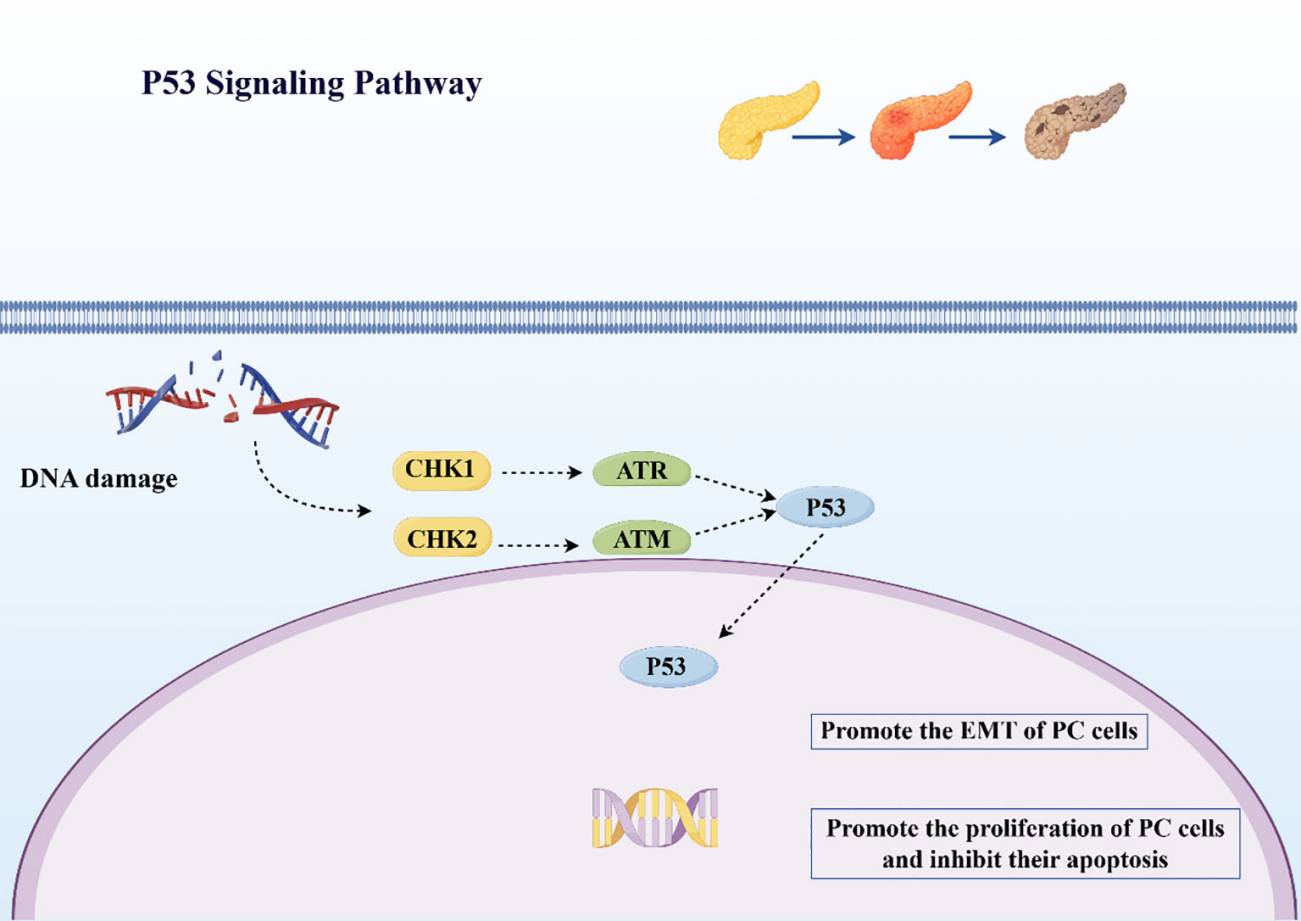
The role of the P53 signaling pathway in the pathogenesis of pancreatic cancer.

In the absence of oncogenic KRAS mutations, deletion of Trp53/Smad4 or Trp53/Tgfbr2 can both induce spontaneous PC formation (127). Studies have found that LINC00857, a lncRNA, is significantly upregulated in PC with TP53 mutations. Atorvastatin, a commonly used lipid-lowering drug, can inhibit PC metastasis via the mutant TP53-LINC00857 axis (128). STAT3 undergoes constitutive phosphorylation in murine PC cells with TP53 mutation or deletion. Genetic ablation of STAT3 in mice, or pharmacological inhibitors targeting JAK2 or STAT3 activation, reduces fibrosis and the number of pancreatic stellate cells in the tumor stroma, and alters the types of immune cells infiltrating the tumor. Loss of TP53 function activates the JAK2-STAT3 signaling pathway, which promotes tumor stromal remodeling, tumor growth, and resistance to gemcitabine (129) (**Figure 7**). Compared with KRAS, the clinical translation of p53 restoration or mutant p53-targeted therapy still faces substantial challenges. Nevertheless, p53 retains considerable research value as a prognostic biomarker, a molecular stratification indicator, and a target for combination therapy.

## 6 Targeted therapy and immunotherapy

### 6.1 KRAS-targeted agents

#### 6.1.1 KRAS G12C inhibitors

Sotorasib is a small-molecule inhibitor targeting the KRAS G12C mutation, which has been used clinically for the treatment of patients with advanced non-small cell lung cancer harboring this mutation (130). Strickler and other scholars (131) reported that 1%–2% of PC patients carry the KRAS G12C mutation. They conducted a phase I–II trial enrolling 38 patients with metastatic pancreatic cancer previously treated with chemotherapy to evaluate the safety and efficacy of sotorasib. The study showed an objective response rate of 21% and a median overall survival of 6.9 months, demonstrating significant antitumor activity. Treatment-related adverse events included liver function abnormalities in 42% of patients, but there were no treatment-related deaths or discontinuations due to adverse events, indicating an acceptable safety profile.

#### 6.1.2 KRAS G12D inhibitors

Approximately half of PC patients harbor the KRAS G12D mutation. Mirati Therapeutics developed MRTX1133, the first non-covalent, potent, and selective KRAS G12D inhibitor (132). Mahadevan and other scholars (133) used 16 different models to confirm that KRAS G12D drives pancreatic carcinogenesis and demonstrated that MRTX1133 reverses early PC growth, increases intratumoral CD8+ effector T cells, reduces myeloid infiltration, and reprograms cancer-associated fibroblasts. Gulay and other scholars (134) showed that low-concentration MRTX1133 exerted highly selective antitumor activity in cell lines and patient-derived organoid models carrying the KRAS G12D mutation. Meanwhile, MRTX1133 exhibited strong synergistic effects with the pan-ERBB inhibitor afatinib *in vitro*. This combination strategy induced tumor regression and prolonged survival in mouse models of PC. As the first KRAS G12D inhibitor to enter clinical development, MRTX1133 validated the druggability of this target and sparked intensive research on KRAS G12D inhibitors.

#### 6.1.3 Pan-KRAS inhibitors

RMC-6236 is an oral pan-KRAS inhibitor with inhibitory activity against multiple KRAS mutant variants. Preclinical studies showed that this compound effectively inhibits the phosphorylation of extracellular signal-regulated kinase, suppresses the proliferation of various PC cell lines *in vitro*, and induces apoptosis (135). RMC-6236 exhibits broad therapeutic potential with antitumor activity across diverse KRAS mutant subtypes. Its combination with afatinib and SD36 induced complete regression of PC and significantly delayed tumor progression in mice. These findings support the clinical development of novel therapeutic strategies for PC patients (136).

### 6.2 Immune checkpoint inhibitors

Currently, the most representative immune checkpoints include programmed death 1 (PD-1) and cytotoxic T lymphocyte-associated antigen-4 (CTLA-4). However, their therapeutic efficacy in pancreatic cancer remains relatively limited.

#### 6.2.1 Anti-CTLA-4 monoclonal antibodies

Ipilimumab is a monoclonal antibody targeting CTLA-4. A phase IIb trial evaluated the efficacy of ipilimumab combined with gemcitabine in advanced PC. A total of 21 patients were enrolled, with an objective response rate of 14%. The combination was considered safe and feasible for advanced PC, but its efficacy was comparable to that of gemcitabine monotherapy (137). PC is resistant to both chemotherapy and immunotherapy, and the CTLA-4 immune checkpoint may represent a therapeutic breakthrough. Further studies are needed to explore combination regimens with other therapies to identify rational treatment strategies for patients.

#### 6.2.2 Anti-PD-1 monoclonal antibodies

Sintilimab is a monoclonal antibody against PD-1 that blocks the interaction between PD-1 and its ligands, thereby restoring the antitumor function of T cells (138). A single-center, randomized, controlled, open-label phase II trial (CISPD-3) (139) randomized 110 PC patients 1:1 to receive sintilimab alone or sintilimab plus modified FOLFIRINOX. The median survival was similar between the two groups (10.9 months vs. 10.8 months), and the primary endpoint was not met in either arm. The benefit of sintilimab combined with modified FOLFIRINOX in patients with advanced PC was not confirmed.

Nivolumab is another PD-1 inhibitor. An open-label, multicenter phase I/II study (140) enrolled 22 patients with advanced PC to evaluate nivolumab combined with stereotactic body radiation therapy. The study suggested that the quality of life was acceptable in patients with locally advanced PC but deteriorated with disease progression. This regimen failed to achieve durable long-term efficacy, highlighting the need for novel therapeutic strategies.

The efficacy of immune checkpoint inhibitors, whether as monotherapy or in combination with other treatments, is unsatisfactory in PC, which may be related to the immune escape mechanisms mediated by the highly complex tumor microenvironment of pancreatic cancer.

## 7 Evidence strength and knowledge gaps

To avoid treating evidence from different levels as equivalent, this review provides the following stratified evaluation of the major mechanisms. This stratification does not represent an absolute hierarchy, but rather a general summary based on the sources of evidence, degree of reproducibility, and clinical translational potential. (**Table 1**)

**Table 1:** Overview of the evidence strength for the etiology and pathogenesis of PC.

Mechanism/factor	Main sources of evidence	Evidence strength	Translational significance	Major limitations
Early KRAS-driven events and PanIN progression	Pathological models, genomic studies, animal models	Strong	High-risk screening, early molecular detection, KRAS-targeted drug development	KRAS alone is insufficient to explain invasiveness and heterogeneity.
IPMN/MCN precursor pathways	Pathological studies, imaging follow-up, genomic evolutionary analyses	Strong to moderate	Surveillance of cystic lesions and determination of surgical timing	Overtreatment of low-risk lesions and identification of high-risk lesions remain challenging.
Smoking	Epidemiological studies, mutagenic mechanisms, animal models	Strong	Primary prevention and smoking cessation interventions	Individual dose–response relationships and gene–environment interactions require further refinement.
Alcohol consumption	Epidemiological studies, pancreatitis-related mechanisms, metabolic injury studies	Moderate	Control of long-term heavy alcohol consumption and pancreatitis risk	Substantial confounding by smoking and lifestyle factors.
Obesity/diabetes	Cohort studies, metabolic mechanisms, preclinical models	Moderate to strong	High-risk population screening and metabolic intervention	New-onset diabetes needs to be distinguished as either a causal factor or an early manifestation.
CAF subtypes and immune exclusion	Single-cell sequencing, spatial analysis, animal models	Moderate	Microenvironmental stratification and combination immunotherapy strategies	Insufficient clinical validation; CAF functions are bidirectional.
Lactate metabolism and lactylation	*In vitro* and animal experiments, retrospective biomarker studies	Preliminary to moderate	Potential for combined metabolic–immune targeting |	Insufficient prospective clinical evidence.

Overall, research on the etiological mechanisms of PC has moved beyond the description of single risk factors and entered a stage of multidimensional integration. The strongest evidence supports pathological progression models, KRAS-driven events, inactivation of major tumor suppressor genes, smoking, chronic pancreatitis, and metabolic-inflammatory risk factors such as diabetes and obesity. Areas with moderate evidence but substantial translational potential include IPMN/MCN risk stratification, histological and molecular testing based on EUS-FNB, CAF subtypes, and immune exclusion. Fields that remain exploratory include lactylation modification, dynamic transitions among CAF subtypes, targeting of specific adipokines, and complex multi-omics predictive models.

Current studies and available evidence indicate that microscopic precancerous lesions such as PanIN are difficult to identify at an early stage using conventional imaging, making it challenging to capture the early progression window in clinical practice. Although KRAS mutations are widespread, they are insufficient to predict invasiveness on their own and need to be interpreted together with tumor suppressor gene alterations, inflammatory status, and microenvironmental features. CAFs can exert both tumor-promoting and tumor-restricting effects, and nonselective stromal depletion may produce counterproductive outcomes. The failure of immunotherapy is not simply attributable to insufficient immune checkpoint expression, but rather to the combined effects of immune exclusion, metabolic suppression, and stromal barriers. Many mechanistic studies are derived from mouse models or *in vitro* systems, and validation in human tissues and clinical cohorts remains insufficient.

PC is a complex disease caused by the interaction of multiple factors. Genetic factors induce the occurrence of PC either through direct action or via crosstalk with environmental and epigenetic factors. Meanwhile, metabolic and inflammatory conditions such as diabetes, obesity, and chronic pancreatitis can alter the local pancreatic microenvironment, making KRAS-driven ADM and PanIN more likely to become stabilized and progress toward invasive carcinoma. In addition, as a highly malignant tumor of the digestive system, PC has a complicated pathogenesis involving the dysregulated modulation of multiple signaling pathways. The occurrence and development of PC is a complex process involving multiple genes and multi-stage progression. Oncogene activation, tumor suppressor gene inactivation, and aberrant regulation of signaling pathways represent the core molecular events underlying PC pathogenesis. For instance, KRAS gene mutations are extremely prevalent in PC; its persistent activation can promote tumor cell proliferation, invasion, and metastasis by regulating signaling pathways including PI3K/Akt/mTOR, Wnt/β-catenin, and NF-κB. In contrast, the inactivation of tumor suppressor genes such as P53 and CDKN2A leads to dysregulation of cell cycle control, thereby accelerating tumor progression.

Compared with traditional descriptive reviews, this study places greater emphasis on the hierarchy of evidence supporting different mechanisms. Smoking, chronic pancreatitis, KRAS mutations, and the PanIN progression model show a high degree of consistency and can be regarded as core evidence in etiological research on PC. In contrast, alcohol consumption, adipokines, lactate metabolism, CAF subtypes, and immune exclusion have clear biological significance, but their sources of evidence and clinical translatability are not entirely equivalent. Therefore, preclinical mechanisms should not be directly equated with mature therapeutic targets.

In terms of translational application, early diagnosis and precision treatment of PC require the integration of risk factors, precursor lesions, histological sampling, and molecular classification. EUS-FNB can obtain histological samples preoperatively, providing a basis for diagnosis before neoadjuvant therapy, genetic testing, CAF subtype assessment, and immune microenvironment analysis. Future research should not focus solely on a single target, but should instead establish a continuous clinical pathway encompassing “high-risk population identification—imaging and endoscopic surveillance—histological/molecular validation—individualized treatment selection.”

In the future, with the deeper integration and application of multi-omics technologies, as well as closer coordination between basic research and clinical practice, the etiology and pathogenesis of PC are expected to be further clarified, thereby providing a more reliable theoretical foundation and translational pathway for early screening, risk prediction, treatment stratification, and prognostic improvement.

## 9 Conclusion

The development of PC results from the combined effects of genetic factors, environmental exposure, metabolic abnormalities, chronic inflammation, precancerous lesion progression, and remodeling of the TME. KRAS-driven ADM/PanIN formation, the precursor pathways of IPMN and MCN, and alterations involving CDKN2A, TP53, and SMAD4 participate in disease progression and currently represent relatively well-established mechanistic axes. Factors such as smoking, chronic pancreatitis, diabetes, and obesity are strongly supported by epidemiological evidence, but their specific molecular bridging mechanisms require further validation. CAF subtypes, immune exclusion, and lactate metabolism provide new perspectives for immunotherapy and microenvironment-targeted strategies in PC; however, further confirmation from human tissue-based evidence and prospective clinical studies is still needed. Future research should be based on evidence stratification and promote the translation of PC research from mechanistic investigation toward high-risk population screening, preoperative histological and molecular classification, and precision combination therapy.

## Glossary

ADM: Acinar-to-ductal metaplasia
apCAFs: Antigen-presenting CAFs
Akt: Akt kinase
ATM: Ataxia-telangiectasia mutated gene
ATR: Ataxia-telangiectasia and Rad3-related gene
BMI: Body mass index
BMP: Bone morphogenetic protein
CAFs: Cancer-associated fibroblasts
Chk1: Checkpoint kinase 1
Chk2: Checkpoint kinase 2
CTLA-4: Cytotoxic T lymphocyte-associated antigen-4
ECM: Extracellular matrix
EGFR: Epidermal growth factor receptor
EMT: Epithelial-mesenchymal transition
EUS-FNB: Endoscopic ultrasound-guided fine-needle biopsy
GSK-3β: Glycogen synthase kinase-3β
GWAS: Genome-wide association study
HB-EGF: Heparin-binding epidermal growth factor-like growth factor
HIF-1α: Hypoxia-inducible factor-1α
HK2: Hexokinase 2
iCAFs: Inflammatory CAFs
IκB: Inhibitor of NF-κB
IGF-1R: Insulin-like growth factor 1 receptor
IKK: Inhibitor of NF-κB kinase
ILs: Interleukins
IPMNs: Intraductal papillary mucinous neoplasms
IRAK2: Interleukin-1 receptor-associated kinase 2
JAK: Janus kinase
LCN2: Lipocalin-2
lncRNA: Long non-coding RNA
LDH: Lactate dehydrogenase
LDHA: Lactate dehydrogenase A
MCN: Mucinous cystic neoplasm
MICA/B: Major histocompatibility complex class I chain-related proteins A and B
mTOR: Mammalian target of rapamycin
MMPs: Matrix metalloproteinases
MMP-9: Matrix metalloproteinase-9
myCAFs: Myofibroblastic CAFs
ncRNA: Non-coding RNA
NK: Natural killer
PanIN: Pancreatic intraepithelial neoplasia
PC: Pancreatic cancer
PCLs: Pancreatic cystic lesions
PD-1: Programmed death 1
PDAC: Pancreatic ductal adenocarcinoma
PFKP: Phosphofructokinase platelet
PIP3: Phosphatidylinositol-3,4,5-trisphosphate
RNF43: Ring finger protein 43
STAT: Signal transducer and activator of transcription
TAMs: Tumor-associated macrophages
TAMs: Tumor-associated macrophages
TCF4: Transcription factor 4
TGF-β: Transforming growth factor-β
TME: Tumor microenvironment
VEGF: Vascular endothelial growth factor
WHO: World Health Organization.
